# Pleuro-pulmonary tumours detected by clinical and chest X-ray analyses in rats transplanted with mesothelioma cells

**DOI:** 10.1038/sj.bjc.6693248

**Published:** 1999-12

**Authors:** F Le Pimpec-Barthes, I Bernard, I Abd Alsamad, A Renier, L Kheuang, J Fleury-Feith, P Devauchelle, F Quintin Colonna, M Riquet, M C Jaurand

**Affiliations:** Service de Chirurgie Thoracique, Hôpital Laennec, 42 rue de Sèvres, Paris 75007, France; Service de Microbiologie, Service de Cancérologie, Ecole Nationale Vétérinaire d’Alfort, Maisons Alfort, 94704, France; Laboratoire d’Anatomo-Pathologie, Centre Hospitalier Intercommunal de Créteil, 40 avenue de Verdun, Créteil Cedex 940010, France; XR139 INSERM et EA2345, IM3, Faculté de Médecine, 8 rue du Général Sarrail, Créteil 94010, Cedex, France; Laboratoire d’Histologie et de Biologie Tumorale, Hôpital Tenon, Paris 75020, France

**Keywords:** culture, malignant mesothelioma, nude rats, transplantation

## Abstract

New strategies for cancer therapy must be developed, especially in severe neoplasms such as malignant pleural mesothelioma. Animal models of cancer, as close as possible to the human situation, are needed to investigate novel therapeutical approaches. Orthotopic transplantation of cancer cells is then relevant and efforts should be made to follow up tumour evolution in animals. In the present study, we developed a method for the orthotopic growth of mesothelioma cells in the pleural cavity of Fischer 344 and nude rats, along with a procedure for clinical survey. Two mesothelioma cell lines, of rat and human origin, were inoculated by transthoracic puncture. Body weight determination and chest X-ray analyses permitted the follow-up of tumour evolution by identifying different stages. Autopsies showed that tumours localized on the whole pleural cavity (diaphragm, parietal pleura), mediastinum and pericardium. Tumour morphology and antigenic characteristics were consistent with those of the inoculated cells and were similar in both types of rats inoculated with the same cell type. These results demonstrate that mesothelioma formation in rats can be followed up by clinical and radiographic survey after gentle intrathoracic inoculation of mesothelioma cells, thus allowing the definition of stages of interest for further experimental trials. © 1999 Cancer Research Campaign


*British Journal of Cancer* (1999) **81**(8), 1344–1350
© 1999 Cancer Research Campaign
Article no. bjoc.1999.0850

### Pleuro-pulmonary tumours detected by clinical and chest X-ray analyses in rats transplanted with mesothelioma cells

**F Le Pimpec-Barthes[1], I Bernard[2], I Abd Alsamad[4], A Renier[5], L Kheuang[5], J Fleury-Feith[6], P Devauchelle[3], F Quintin Colonna[2], M Riquet[1] and MC Jaurand[5]**

[1]Service de Chirurgie Thoracique, Hôpital Laennec, 42 rue de Sèvres, 75007 Paris, France; [2]Service de Microbiologie et [3]Service de Cancérologie, Ecole Nationale Vétérinaire d'Alfort, 94704, Maisons Alfort, France; [4]Laboratoire d'Anatomo-Pathologie, Centre Hospitalier Intercommunal de Créteil, 40 avenue de Verdun, 940010 Créteil Cedex, France; [5]XR139 INSERM et EA2345, IM3, Faculté de Médecine, 8 rue du Général Sarrail, 94010 Créteil, Cedex, France; [6]Laboratoire d'Histologie et de Biologie Tumorale, Hôpital Tenon, 75020 Paris, France

**Summary** New strategies for cancer therapy must be developed, especially in severe neoplasms such as malignant pleural mesothelioma. Animal models of cancer, as close as possible to the human situation, are needed to investigate novel therapeutical approaches. Orthotopic transplantation of cancer cells is then relevant and efforts should be made to follow up tumour evolution in animals. In the present study, we developed a method for the orthotopic growth of mesothelioma cells in the pleural cavity of Fischer 344 and nude rats, along with a procedure for clinical survey. Two mesothelioma cell lines, of rat and human origin, were inoculated by transthoracic puncture. Body weight determination and chest X-ray analyses permitted the follow-up of tumour evolution by identifying different stages. Autopsies showed that tumours localized on the whole pleural cavity (diaphragm, parietal pleura), mediastinum and pericardium. Tumour morphology and antigenic characteristics were consistent with those of the inoculated cells and were similar in both types of rats inoculated with the same cell type. These results demonstrate that mesothelioma formation in rats can be followed up by clinical and radiographic survey after gentle intrathoracic inoculation of mesothelioma cells, thus allowing the definition of stages of interest for further experimental trials. © 1999 Cancer Research Campaign

**Keywords:** culture; malignant mesothelioma; nude rats; transplantation

Experimental induction of tumours has been widely used to investigate the anti-tumoural action of agents, which are candidates for therapeutical strategies. Despite some limitations, these methods offer useful tools, at a preclinical stage, in order to obtain information on the action mechanism of antineoplastic drugs and to investigate tumour regression. Transplantation of tumour fragments and isolated cells is currently used; subcutaneous (s.c.) inoculation of tumour cells in nude mice is the most common and easy method for growing tumours from human neoplastic cells (Baselga and Mendelsohn, 1994; Bishop, 1995; Davis et al, 1996; Perrin et al, 1997). Severe combined immune deficient (SCID) mice also show tumour cell proliferation. According to Chahinian et al (1991), xenografts in nude mice are relevant models to investigate a therapeutical potency in specific tumours. To improve the relevance of these methods, orthotopic transplantations have been performed (Astoul et al, 1993; Togo et al, 1995).

Recently, a great deal of interest has focused on human malignant mesothelioma, regarding the severity of the disease, its poor prognosis (Hoogsteden et al, 1997), and an increasing number of cases associated with past asbestos exposure and other unknown reasons, possibly involving SV40 contamination (Carbone et al, 1997). In addition to s.c. implantation, several other models have been employed including pleural grafts in immunodeficient mice or rats, and intraperitoneal (i.p.) injection of tumour cells (Astoul et al, 1993; Smythe et al, 1994; Esandi et al, 1997). However, tumour implantation through surgery entails inflammatory reactions, scarring and local fibrosis that may interfere with tumour evolution. Despite its interest regarding similarities between the pleural and peritoneal environment, the intraperitoneal site is not the most appropriate for growing mesothelioma cells because peritoneal mesothelioma is less frequent than pleural mesothelioma in humans. It is therefore interesting to develop models as close as possible to the human situation in order to mimic the human disease and to follow up both tumour growth and regression when the models have to be used for therapeutic investigations. This latter approach requires suitable methods to determine the clinical status of the animals in order to assess the efficiency of the treatment without sacrifice.

To address these issues we developed a non-aggressive method for orthotopic transplantation of rat mesothelioma cells in syngenic and nude rats, and human mesothelioma cells in nude rats, and followed the post-transplantation clinical evolution. We demonstrate that tumour evolution can be evaluated by chest X-ray and body weight.

### MATERIALS AND METHODS

### Mesothelioma cells

Rat mesothelioma cells were grown from pleural mesothelioma developed in Fischer 344 rats following the intrapleural

*Received 3 September 1998*
*Revised 12 January 1999*
*Accepted 27 January 1999*

*Correspondence to:* MC Jaurand inoculation of 20 mg of crocidolite fibres. The protocols for fibre inoculation, rat housing and survey, and tumour harvesting have been described elsewhere (Jaurand et al, 1987). Crocidolite fibres were provided by the Union Internationale Contre le Cancer. After tumour excision, mesothelioma cells were cultured according to standard conditions in RPMI-1640 (Gibco BRL, Life Technologies, Cergy Pontoise, France) supplemented with 8% fetal bovine serum (FBS; Biological Industries, ATGC, Noisy le Grand, France), penicillin and streptomycin, both from Gibco BRL as described elsewhere (Fleury-Feith et al, 1989). After several passages to obtain a sufficient number of cells, these were frozen in complete RPMI-1640 containing 10% dimethyl sulphoxide (Sigma, Saint Quentin Fallavier, France) and stored in liquid nitrogen. The mesothelial origin of the cells was confirmed by the co-expression of keratin and vimentin using an alkaline phosphatase anti-alkaline phosphatase (APAAP) method (Zeng et al, 1994). The antibodies used were polyclonal rabbit antibodies directed against human 56-kDa cytokeratin and monoclonal mouse anti-swine vimentin 57-kDa, both obtained from DAKO (78196 Trappes, France).

A human mesothelioma cell line (DEVy) established in our laboratory was also investigated. This cell line exhibited an epithelial morphology and co-expressed keratin and vimentin as previously described (Zeng et al, 1994). In a preliminary study, we found that DEVy formed tumours in nude mice 1 week after the s.c. inoculation of $3 \times 10^6$ cells (unpublished data).

### Cell transplantation method

Two sorts of rats were investigated: Fischer 344 IOPS rats, 5 weeks old were obtained from Iffa Credo (L'Arbresle, France) and nude rats (HsdHan: NZNU-nu, 4–6 weeks old, from Harlan, France). Procedures for housing and handling of animals were in agreement with the European Decret (19 October 1987). The animals were housed for at least 1 week prior to mesothelioma cell inoculation. Rats were anaesthetized by the intramuscular (i.m.) inoculation of a mixture composed of 5 mg $kg^{-1}$ xylazine (ND Rompun 2%) (Bayer Pharma, Puteaux, France) and 40 mg $kg^{-1}$ of Ketamin (ND Imalgene, Rhône Mérieux, Lyon, France). The method used to permit orthotopic growth of mesothelioma cells consisted in the deposition in the pleural cavity of a cell suspension derived from the procedure developed to inject asbestos fibres in the pleural cavity (Monchaux et al, 1981). The desired amount of mesothelioma cells was suspended in 0.3 ml of phosphate-buffered saline (PBS) and sucked into a 1 ml syringe, then fitted to a 0.8 mm diameter needle. The administration was made on the right side in the middle of the thorax, between the 6th and 7th ribs. In a first series of assays, 0.4 ml of a contrast solution (Telebrix 35, 350 mg I $ml^{-1}$; Guerbet, Aulnay sous Bois, France) diluted 3:4 in physiological saline or mesothelioma cells mixed with a contrast solution were administered in order to check the location of the suspension. Chest X-ray radiographs confirmed the intrapleural administration of the solution without lung damage as illustrated in Figure 1.

### Experimental protocol

Rat mesothelioma cells were inoculated in three groups of six Fischer 344 rats treated with $5 \times 10^6$ (group I), $3 \times 10^6$ (group II) or $1 \times 10^6$ (group III) rat mesothelioma cells, according to the procedure described above, without contrast solution. This procedure

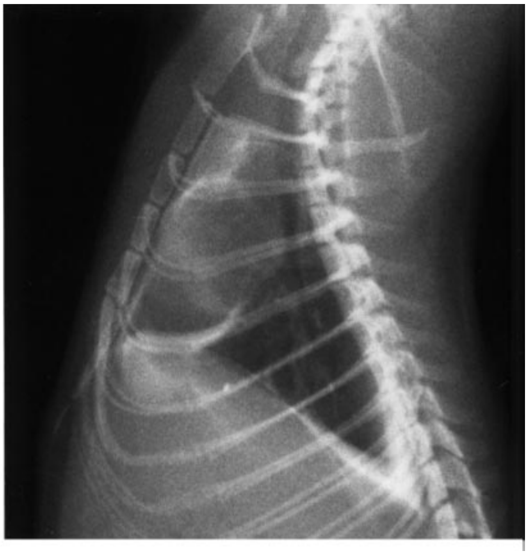

**Figure 1** Chest radiograph from a Fischer 344 rat immediately after intrapleural administration of rat mesothelioma cells suspended in PBS supplemented with contrast liquid solution. This solution diffuses in all the pleural cavity mainly in the posterior slope space. The cardiac opacity is outlined by the solution allowed the determination of the effect of cell concentration and the convenient amount for inoculation in nude rats. Consequently, 11 nude rats were further inoculated with $3 \times 10^6$ rat mesothelioma cells under the same conditions as Fischer 344 rats. In this latter experiment, four sham nude rats received 0.3 ml of PBS. The human mesothelioma cells, DEVy, were inoculated in three nude rats according to the same procedure.

The animal body weight was controlled every week and chest radiographs (Konica Medical Films, MG-SR super rapid) were performed approximately every 10 days, from the second week after treatment. For this purpose, rats were anaesthetized according to the procedure described above. Both face and profile radiographs were made, with a SEDETAL (APR-VET.FL.VET Veterinary Systems H.F. series generator) apparatus under the following conditions: 100 mA, 48 kv, 0.01 s.

The animal's death was either spontaneous or due to a decision of euthanasia because of body weight loss. Autopsies were carried out on all subjects. Radiograph interpretations were made blindly before autopsies. A score from 0 to 3 was attributed to the radiographs, depending on the extent of the chest X-ray abnormalities based on mediastinum widening, loss of cardiac silhouette with subcardiac clear space filling and mass deformity of the diaphragm area. A semi-quantification was made in order to perform further therapeutical treatments at different stages of tumour development. The following classifications were made: no abnormality (score 0); localized abnormalities with small tumour volume (score 1); diffuse abnormalities or medium tumour volume (score 2); and large abnormalities with large tumour volume (score 3).

The tumours were fixed in 10% formal in for anatomo-pathological examination or in glutaraldehyde in cacodylate buffer for electron microscopy studies, according to standard procedures (Fleury-Feith et al, 1989). Immunohistochemistry of tumours was performed to investigate keratin expression. Tumours formed after the inoculation of rat mesothelioma cells were incubated with rabbit polyclonal antibodies raised against bovine keratin using monoclonal mouse anti rabbit IgG as secondary antibodies. Both

© Cancer Research Campaign 1999

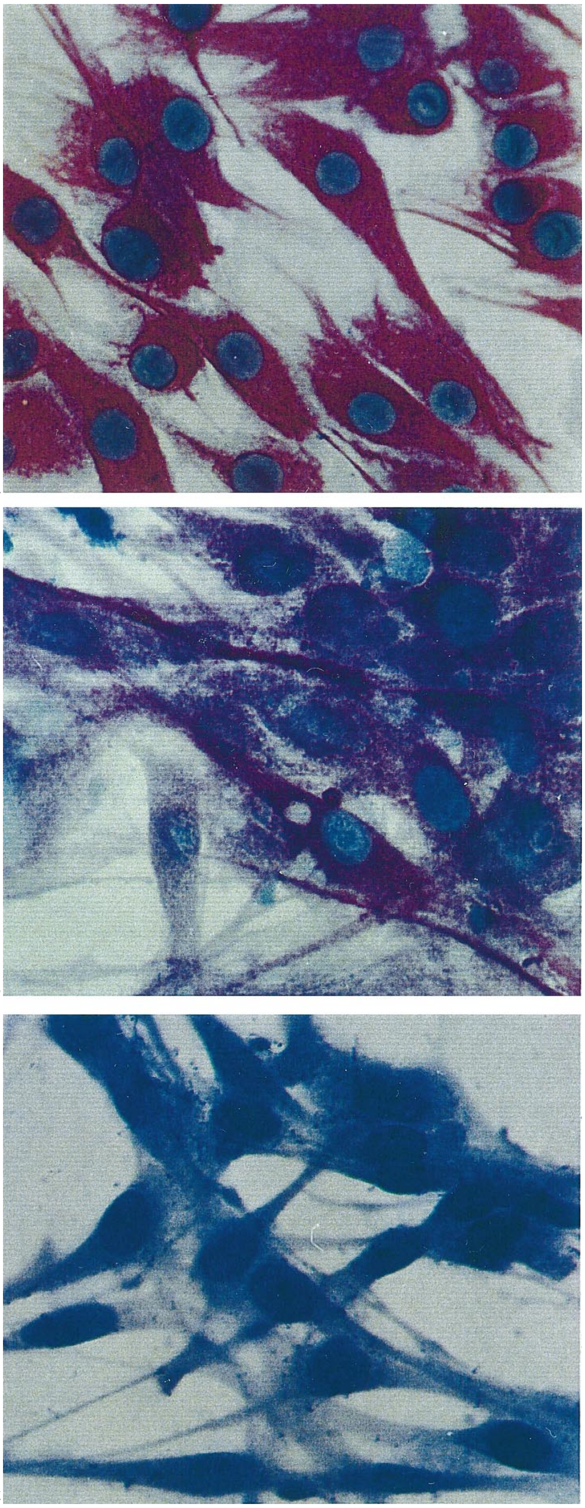

**Figure 2** Micrograph of culture of rat mesothelioma cells obtained from an asbestos-induced rat pleural mesothelioma. Immunostaining with antibodies directed against (**A**) cytokeratin and **(B)** vimentin; **(C)** control without primary antibodies (original magnification × 100).

agents were obtained from DAKO (Trappes, France). The revelation was made with a DAKO LABS® (labelled streptavidin–biotin) kit labelled with alkaline phosphatase. Vimentin expression was determined with a DAKO clone V9 antibodies followed by a peroxidase revelation.

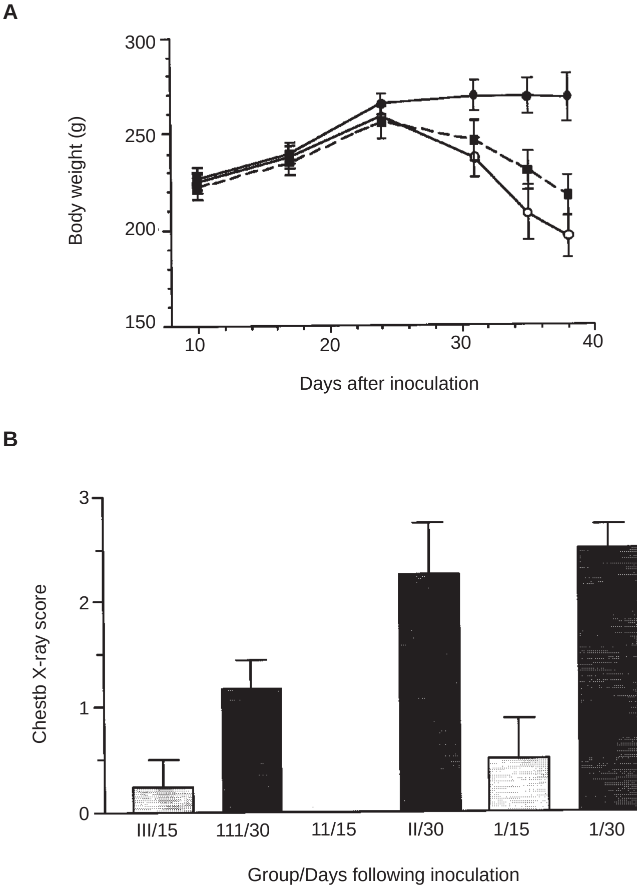

**Figure 3 (A)** Kinetics of body weight of Fischer 344 rats inoculated with rat pleural mesothelioma cells: Group I (–○–); Group II (– –■– –); Group III (–●–).**(B)** Chest X-ray scores (mean ± s.e.m.), 15 days (■) and 30 days (■) after inoculation with mesothelioma cells

### Statistical analyses

Analysis of variance (ANOVA) analyses and *t*-tests were performed for weight and chest X-rays score comparisons (GraphPad PRISM® software, V 2.0 for Macintosh).

### RESULTS

### Characterization of the rat mesothelioma cell line

We first investigated the phenotype of the cells cultured from asbestos-induced pleural tumours in Fischer 344 rats used for transplantation in the present study. This cell line exhibited a fusiform phenotype but coexpressed keratin and vimentin. Figure 2 shows the morphology and antigenic characterization.

### Clinical studies

Figure 3A reports the mean weight of Fischer 344 rats in the different groups. In all groups, the mean body weight increased up to about 3 weeks after intrapleural administration of tumour cells. This increase was normal since these rats continued to gain weight linearly up to 10 weeks. Thereafter their weight continued to increase slowly. In the treated animals, a stabilization in weight rise was observed after 3 weeks in groups III, indicating some

© Cancer Research Campaign 1999

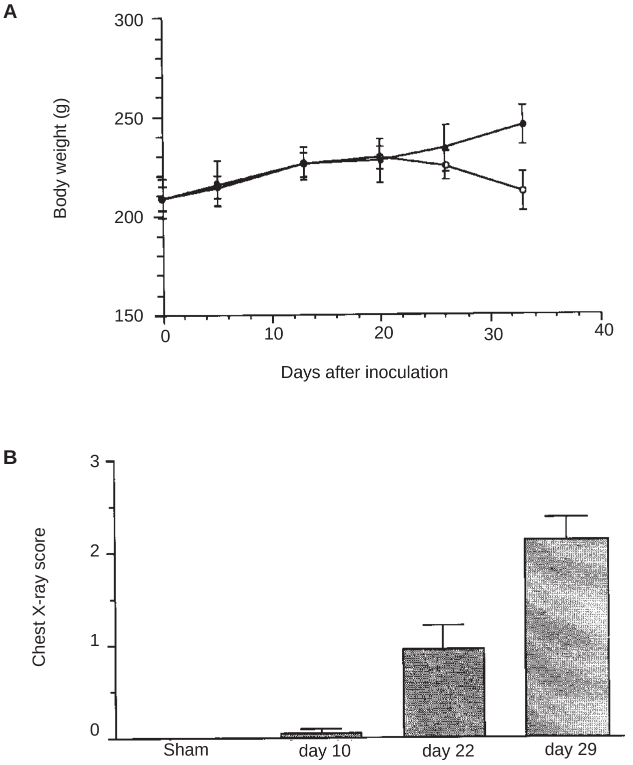

**Figure 4** (**A**) Kinetics of body weight of nude rats inoculated with rat pleural mesothelioma cells: Sham-treated (–○–); treated with mesothelioma cells (–■–). **(B)** Chest X-ray scores in rats inoculated with mesothelioma cells (■); mean ± s.e.m.

metabolic disturbances. Thereafter, a weight loss was evident in groups I and II, occurring between 24 and 31 days after inoculation. No obvious decrease was observed in the rats belonging to group III. Statistical analyses showed significant body weight was found in two group ($P < 10^{-4}$, ANOVA). Moreover, a significant decrease in body weight was found in two groups: a significant lowering in body weight was detected between 24 and 31 days in group II (paired *t*-test: $P = 0.044$) and in group I ($P = 0.015$); no significant change was observed in group III ($P = 0.73$).

In order to determine whether the response of nude rats differed from that observed in immunocompetent animals, we inoculated nude rats with the same cell line, under similar conditions with $3 \times 10^6$ cells. Figure 4A reports the body weight evolution of nude rats. All rats continued to gain weight until 13 days after treatment, but the body weight of rats inoculated with mesothelioma cells was reduced after 3 weeks post-inoculation. Significant time-dependent body weight changes were found in both sham and treated rats ($P < 10^{-4}$ ANOVA). The body weight of sham animals continued to rise between 20 and 33 days post-inoculation (paired *t*-test: $P = 0.006$). In contrast, the body weight of treated animals decreased at the same time (paired *t*-test: $P = 0.0096$). However, the differences in body weight in mesothelioma cells versus sham-treated animals was not statistically significant. Despite the limited number of nude rats inoculated with the human mesothelioma cell line, similar conclusions can be drawn. A reduction in body weight of 7% to 3%, depending on the animal, was observed 20 days after inoculation (data not shown).

### Chest X-ray analyses

Figure 5 illustrates the radiographical abnormalities encountered in treated animals. They consisted in pleural effusion, widening of the mediastinal opacity and more or less filling of clear space under cardiac opacity. Table 1 reports the radiographic scores attributed 15 and 30 days following intrapleural administration of the rat pleural mesothelioma cell line in Fischer 344 rats. In no group were important abnormalities detected 15 days after inoculation of rat mesothelioma cells, but differences among the three groups appeared later. The mean scores were 0.25, 0 and 0.5 in groups III, II and I, repectively, after 15 days; they reached 1.2, 2.25 and 2.5 after 30 days (Figure 3B). There was a significant enhancement in chest X-ray scores between 15 and 30 days in groups II and I ($P = 0.005$ and 0.0012 respectively, *t*-test) but not in group III for $\alpha = 5\%$ ($P = 0.051$). Features suggesting massive tumour volumes were observed in all rats from group I and in most of the rats in group II 30 days after inoculation. In these groups, tumour progression was observed in the surviving rats, on chest X-rays performed 43 days after inoculation since all scores reached 3. The tumour progression, as assessed by chest X-ray analysis, was also observed in rats from group III at the end of the experiment (day 43) but the radiographic changes were more limited after 30 days, in comparison with the other groups since the mean value was lower than 2 (Figure 3B) and no individual score reached 3.

Table 2 reports the chest X-ray analyses in nude rats inoculated with rat mesothelioma cells 10, 22 and 29 days after inoculation. Chest X-ray abnormalities were detected 22 days after inoculation in seven out of 11 rats inoculated with the rat mesothelioma cells. After a delay of 29 days, scores were higher than, or equal to, 1 in every rat while normal chest X-rays were observed in sham animals. Scores were significantly enhanced between 10 and 22 days after inoculation ($P = 0.0023$).

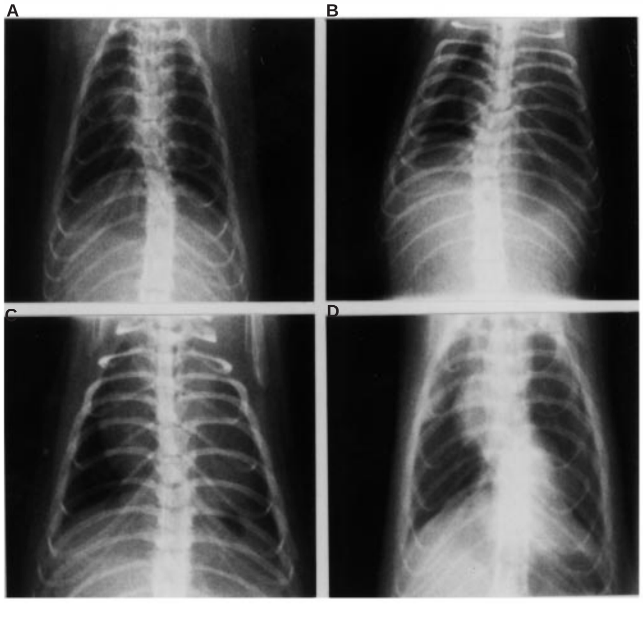

**Figure 5** Chest X-ray films showing: normal chest X-rays **(A)**; pleural effusion in all the cavity **(B)**; widening of the mediastinal opacity **(C)**; filling clear space under cardiac opacity **(D)**

**Table 1** Chest X-ray analysis of Fischer 344 rats after intrapleural administration of a rat pleural mesothelioma cell line

| Group | Rat no. | Delay after inoculation (days) | |
|---|---|---|---|
| | | 15 | 30 |
| I | 1 | 0–1 | 3 |
| | 2 | 0 | 2 |
| | 3 | 0 | 3 |
| | 4 | 0 | 3 |
| | 5 | ND | 2 |
| | 6 | ND | 2 |
| II | 7 | 0 | 3 |
| | 8 | 0 | 3 |
| | 9 | 0 | 1 |
| | 10 | 0 | 3 |
| | 11 | ND | 3 |
| | 12 | ND | 0–1 |
| III | 13 | 0 | 0–1 |
| | 14 | 1 | 2 |
| | 15 | 0 | 1 |
| | 16 | 0 | 1 |
| | 17 | ND | 2 |
| | 18 | ND | 0–1 |

ND: not done.

**Table 2** Chest X-ray analysis of nude rats after intrapleural administration of a rat pleural mesothelioma cell line*

| Group | Rat no. | Delay after inoculation (days) | | |
|---|---|---|---|---|
| | | 10 | 22 | 29 |
| Treated | 1a | 0 | 0 | 1–2 |
| | 1b | 0 | 2 | 3[a] |
| | 2a | 0 | 0 | 2 |
| | 2b | 0 | 2 | 3 |
| | 2c | 0 | 0 | 1 |
| | 3c | 0 | 0 | 1 |
| | 4a | 0 | 1 | 2 |
| | 4b | 0–1 | 2 | 3 |
| | 5 | 0 | 1 | 2 |
| | 6 | 0 | 1–2 | 2 |
| | 7 | 0 | 1 | 3[a] |
| Sham | 3a | 0 | 0 | 0 |
| | 3b | 0 | 0 | 0 |
| | 8 | 0 | 0 | 0 |
| | 9 | 0 | 0 | 0 |

[a]Pleural fluid at autopsy.

### Pathological findings

### *Gross findings*

Autopsies were performed in three Fischer 344 rats after spontaneous death, and after euthanasia decided on the basis of body weight loss and chest X-ray abnormalities in the remaining animals. All rats exhibited massive tumours predominantly invading the mediastinum and pericardium and involving the diaphragm (Figure 6). An extension to the lungs was occasionally found. Tumour metastases were not found in the visceral organs. Similar aspects were observed with human cells and rat cells.

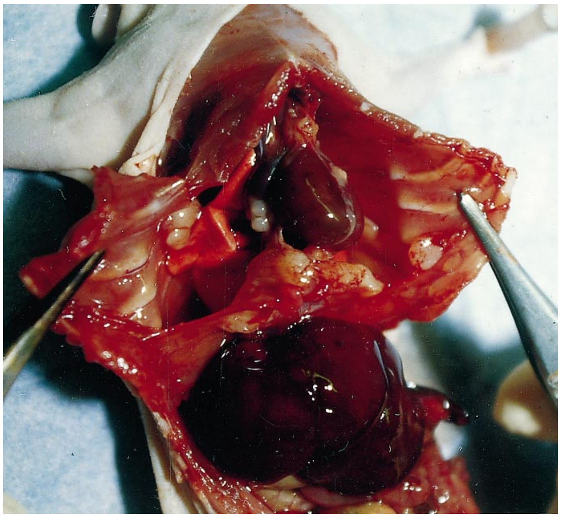

**Figure 6** Autopsy from a nude rat inoculated with rat mesothelioma cells. Gross examination showing nodules are at different locations on the parietal pleura, under cardiac space and on the cardiac pleura

### *Anatomo-pathology of tumours*

Pleural tumours that developed after implantation of rat mesothelioma cells in Fischer 344 rats were examined according to routine protocols. The semi-thin sections demonstrated a fusiform tumour phenotype with infiltration of mastocytes and in some cases tumour invasion in adjacent tissues. A few lymphocytes infiltrated the tumour. According to ultrastructural analysis, a proliferation of cells rich in ribosomes and rough endoplasmic reticulum was detected. In some cases the presence of desmosomes provided a clue for epithelial differentiation of the cells. Immuno-histochemistry analysis demonstrated the expression of keratin and vimentin in the tumour cells (Figure 7). Similar features were found in Fischer 344 rats and in nude rats. Tumours formed in nude rats inoculated with the human mesothelioma cell line were consistent with epithelial malignant mesothelioma.

### DISCUSSION

The data reported in this study have demonstrated that orthotopic mesothelioma growth can be obtained by a careful administration of tumour cells within the pleural space in anaesthetized rats, without surgical trauma. In addition, we found that chest X-ray analysis can be carried out to detect pleural tumours. To our knowledge, this is the first report on a systematic investigation of clinical survey and tumour evolution in rats.

As far as pleural and lung tumours are concerned, tumour transplantation has generally been carried out by s.c. injection of neoplastic cells in immunodeficient mice, or to perfect a more relevant model of mesothelioma, in the peritoneal cavity of mice (Smythe et al, 1994; Hwang et al, 1995). More recently, orthotopic implantation by injection via thoracotomy (Astoul et al, 1993; Esandi et al, 1997) has been developed providing a relevant model of pleural mesothelioma. However, thoracotomy is invariably associated with trauma to the pleural cells and subpleural tissue then followed by processes of inflammation and tissue repair. The production of inflammatory molecules and growth factors may interact with neoplastic cell proliferation and graft. In the present study the use of delicate transthoracic inoculation will minimize

© Cancer Research Campaign 1999

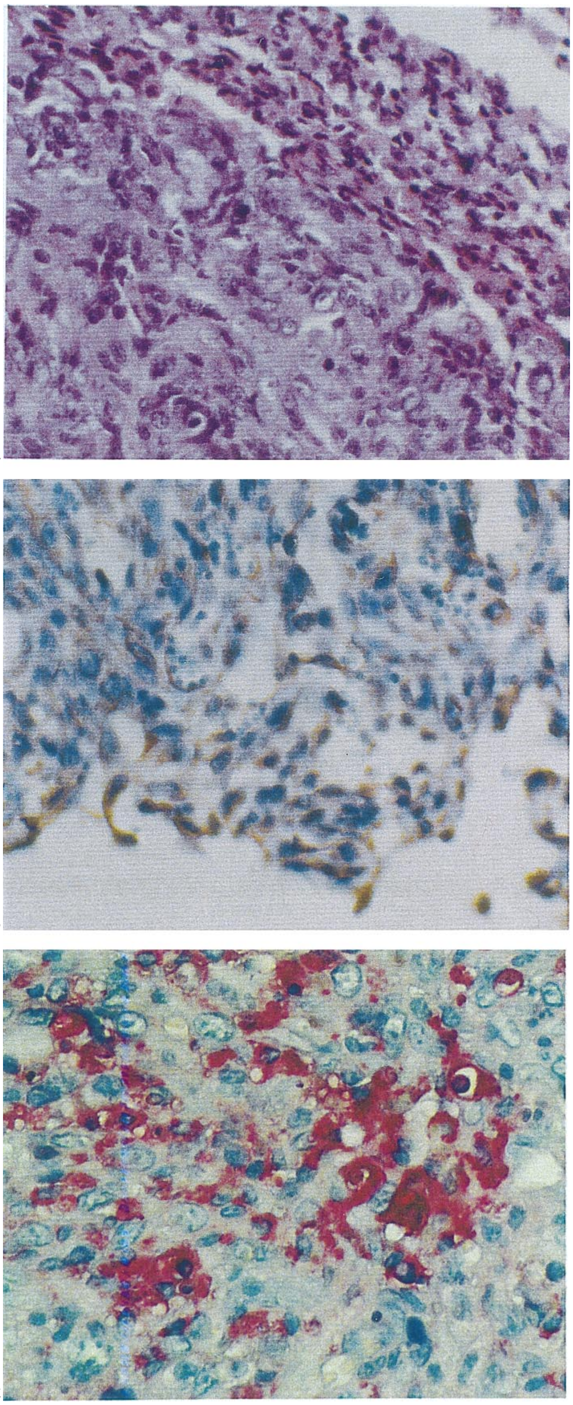

**Figure 7** Typical histological section of a tumour found in Fischer 344 rats inoculated with rat mesothelioma cells. (**A**) Hematoxylin and eosin stain; immunostaining with antibodies directed against (**B**) vimentin, (**C**) keratin (original magnification × 400)

these reactions (Gutman and Fidler, 1995). A pitfall of this method could occur in the damage to lung parenchyma and/or creation of pneumothorax due to needle injury. However, these problems were never encountered in our experiments.

The data reported here have demonstrated that cells isolated from pleural malignant mesothelioma produced in Fischer 344 rats following inoculation of asbestos fibres can be successfully transplanted in both syngenic and nude rats, and that human mesothelioma cells formed malignant mesothelioma when xenografted in nude rats. There was a clear-cut dose–response effect when several concentrations of cells were inoculated. Chest X-ray analyses confirmed the occurrence of the disease and permitted the detection of pulmonary and pleural abnormalities in correlation with the clinical status. The body weight curves appear to be a good clinical parameter to detect mesothelioma growth. In Fischer 344 rats a weight loss was associated with a rapid progression of the disease, as assessed by chest X-ray analysis and confirmed by autopsies.

Autopsies in Fischer rats that died spontaneously have shown that the tumour grew in the pleural cavity and diffused to the lung, but no metastases were found despite a massive invasion of the lung in later stages. Therefore, animal death was clearly associated with the respiratory malignancy. The features are similar to those found in the human situation (Corson, 1986). Similar patterns were observed in both euthanased Fischer 344 and nude rats inoculated with rat mesothelioma cells.

Mesotheliomas formed after the inoculation of the human mesothelioma cells were less invasive, but nodules were found at the same place as in syngenic rats. In addition, some adherence between the diaphragm and liver was detected in nude rats inoculated with human mesothelioma cells.

Tumour histology was in agreement with the cytological findings exhibited by the cultured cells, that is poorly differentiated tumours composed of fusiform cells co-expressing keratin and vimentin in the case of mesothelioma resulting from the inoculation of rat cells, and epithelial mesothelioma after inoculation of the human mesothelioma cells. It may be emphasized that the histological features of the tumours were similar in every animal, independently of the rat strain and of the stage of the disease. This reproducibility suggests that large groups of animals should not be necessary for experimental trials.

It was also found that rat mesothelioma cell transplantation in Fischer 344 rats resulted in faster tumour growth in comparison with that of nude rats. Thirty days after inoculation, two out of three chest X-ray films in Fischer 344 rats reached a score of 3, while only 37% of the nude rats reached that score at the same period. However, gross pathological findings and morphological features of resulting tumours were similar in both sorts of rats, indicating that nude rats develop tumours similar to those formed in immunocompetent rats. This observation is very important regarding the need to use immunodeficient rats for transplantation of human cells. We can therefore suggest that the pathological features of tumours formed in nude rats will have some relevance to human pathology.

Nowadays, it is important to have accurate models to test new therapeutical approaches and understand the action mechanism of drugs at the cellular and molecular level, especially regarding the development of new strategies, including gene transfer in tumours as recently recommended by the National Institutes of Health (Ross et al, 1996). Animal models are therefore necessary to achieve this goal. The model developed here will be useful to investigate mesothelioma proliferation and the effects of drugs and new strategies of interest for mesothelioma therapy. Our studies also demonstrated that the clinical survey of rats is possible. This is an important finding because it will permit both the follow-up of tumour evolution and regression whilst keeping the animal alive and the observation of a recurrence of the disease. Sterman et al (1993) recently reported results of a phase I clinical trial using adenovirus-mediated herpes simplex virus thymidine

*© Cancer Research Campaign 1999* 

kinase/ganciclovir gene therapy in malignant mesothelioma. The authors demonstrated that improvement of these methods is necessary for a better transfer of the vector in the tumours. The use of animal models is therefore of paramount importance to investigate the extent and depth of the virus in the tumour. The present experiments have demonstrated that gentle administration of mesothelioma cells in the rat pleural cavity and clinical survey are relevant and useful tools for malignant mesothelioma investigation in immunocompetent as well as in immunodeficent rats.

### ACKNOWLEDGEMENTS

This study has been carried out with funding from INSERM and Université Paris Val-de-Marne. The authors thank the Ligue Nationale contre le Cancer, Comité du Val d'Oise and their subscribers for providing grants to carry out the research, Dr I Monnet, Service de Pneumologie, CHI Créteil for providing human mesothelioma samples (Zeng et al, 1994), and Prof. D Begon and M Pozetto from the Radiology Unit at Ecole Nationale Vétérinaire d'Alfort for performance of chest X-rays.

### REFERENCES

Astoul P, Viallat JR, Laurent JC, Brandely M and Boutin C (1993) Intrapleural recombinant IL-2 in passive immunotherapy for malignant pleural effusion. *Chest* **103**: 209–213

Baselga J and Mendelsohn J (1994) The epidermal growth factor receptor as a target for therapy in breast cancer. *Breast Cancer Res Treat* **29**: 127–138

Bishop JF (1995) Scheduling of chemotherapy in locally advanced non-small-cell lung cancer. *Lung Cancer* **12**: S53–S61

Carbone M, Rizzo P and Pass HI (1997) Simian virus 40, poliovaccines and human tumors: a review of recent developments. *Oncogene* **15**: 1877–1888

Chahinian AP, Kirschner PA, Gordon RE, Szrajer L and Holland JF (1991) Usefulness of the nude mouse model in mesothelioma based on a direct patient-xenograft comparison. *Cancer* **68**: 558–560

Corson JM (1986) Pathology of malignant mesothelioma. In: *Asbestos-Related Malignancy*. Antman K and Aisner J (eds), pp. 179–199. Harcourt Brace Jovanovich: New York

Davis BM, Koc ON, Lee K and Gerson SL (1996) Current progress in the gene therapy of cancer. *Curr Opin Oncol* **8**: 499–508

Esandi MC, van Someren GD, Vincent AJPE, van Bekkum DW, Valerio D and Bout A (1997) Gene therapy of experimental malignant mesothelioma using adenovirus vectors encoding the HSVtk gene. *Gene Ther* **4**: 280–287

Fleury-Feith J, Nebut M, Saint-Etienne L, Laurent P, Pinchon MC, Kheuang L, Renier A and Jaurand MC (1989) Occurrence and morphology of tumors induced in nude mice transplanted with chrysotile-transformed rat pleural mesothelial cells. *Biol Cell* **65**: 45–50

Gutman M and Fidler IJ (1995) Biology of cancer colon metastasis. *World J Surg* **19**: 226–234

Hoogsteden HC, Langerak AW, van der Kwast TH, Versnel MA and van Gelder T (1997) Malignant pleural mesothelioma. *Crit Rev Oncol Hematol* **25**: 92–126

Hwang HC, Smythe WR, Elshami AA, Kucharczuk JC, Amin KM, Williams JP, Litzky LA, Kaiser LA and Albelda SM (1995) Gene therapy using adenovirus carrying the herpes simplex-thymidine kinase gene to treat in vivo models of human malignant mesothelioma and lung cancer. *Am J Respir Cell Mol Biol* **13**: 7–16

Jaurand MC, Fleury J, Monchaux G, Nebut M and Bignon J (1987) Pleural carcinogenic potency of mineral fibers (asbestos, attapulgite) and their cytotoxicity on cultured cells. *J Natl Cancer Inst* **79**: 797–804

Monchaux G, Bignon J, Jaurand MC, Lafuma J, Sebastien P, Masse R, Hirsch A and Goni J (1981) Mesothelioma in rats following inoculation with acid-leached chrysotile asbestos and other mineral fibres. *Carcinogenesis* **2**: 229–236

Perrin D, Halazy S and Hill B (1997) Inhibitors of *ras* farnesylation: tomorrow's anticancer agents? *Bull Cancer* **84**: 635–642

Ross G, Erickson R, Knorr D, Motulsky AG, Parkman R, Samulski J, Strauss SE and Smith BR (1996) Gene therapy in the United States: a five-year status report. *Hum Gene Ther* **7**: 1781–1790

Smythe WR, Kaiser LR, Hwang HC, Amin KM, Pilewski JM, Eck SJ, Wilson JM and Albelda SM (1994) Successful adenovirus-mediated gene transfer in an in vivo model of human malignant mesothelioma. *Ann Thorac Surg* **57**: 1395–1401

Sterman DH, Treat J, Litzky LA, Amin KM, Coonrod L, Molnar-Kimber K, Recio A, Knox L, Wilson JM, Albelda SM and Kaiser LR (1998) Adenovirus-mediated herpes simplex virus thymidine kinase/ganciclovir gene therapy in patients with localized malignancy: results of a phase I clinical trial in malignant mesothelioma. *Hum Gene Ther* **9**: 1083–1092

Togo S, Shimada H, Kubota T, Moossa AR and Hoffman RM (1995) Host organ specifically determines cancer progression. *Cancer Res* **55**: 681–684

Zeng L, Fleury-Feith J, Monnet I, Boutin C, Bignon J and Jaurand MC (1994) Immunocytochemical characterization of cell lines from human malignant mesothelioma; characterization of human mesothelioma cell lines by immunocytochemistry with a panel of monoclonal antibodies. *Hum Pathol* **25**: 227–234

© Cancer Research Campaign 1999
